# An *Arabidopsis* neutral ceramidase mutant *ncer1* accumulates hydroxyceramides and is sensitive to oxidative stress

**DOI:** 10.3389/fpls.2015.00460

**Published:** 2015-06-19

**Authors:** Jian Li, Fang-Cheng Bi, Jian Yin, Jian-Xin Wu, Chan Rong, Jia-Li Wu, Nan Yao

**Affiliations:** ^1^State Key Laboratory of Biocontrol, Guangdong Key Laboratory of Plant Resources, School of Life Sciences, Sun Yat-sen University, GuangzhouChina; ^2^Institute of Fruit Tree Research, Guangdong Academy of Agricultural Sciences, GuangzhouChina

**Keywords:** *Arabidopsis* neutral ceramidases, oxidative stress, sphingolipids, hydroxyceramides, ceramides

## Abstract

Ceramidases hydrolyze ceramide into sphingosine and fatty acids and, although ceramidases function as key regulators of sphingolipid homeostasis in mammals, their roles in plants remain largely unknown. Here, we characterized the *Arabidopsis thaliana* ceramidase *At*NCER1, a homolog of human neutral ceramidase. *At*NCER1 localizes predominantly on the endoplasmic reticulum. The *ncer1* T-DNA insertion mutants had no visible phenotype, but accumulated hydroxyceramides, and showed increased sensitivity to oxidative stress induced by methyl viologen. Plants over-expressing *AtNCER1* showed increased tolerance to oxidative stress. These data indicate that the *Arabidopsis* neutral ceramidase affects sphingolipid homeostasis and oxidative stress responses.

## Introduction

An important component of eukaryotic membranes, sphingolipids also function as key signaling molecules to regulate cell differentiation and death (Dickson and Lester, 2002; Hannun and Obeid, 2002; Spiegel and Milstien, 2003; Futerman and Hannun, 2004; Pyne et al., 2009). Genetic and biochemical analyses have identified several enzymes in the sphingolipid pathway. For example, *accelerated cell death 5* (*acd5*) a ceramide kinase deficient mutant (Liang et al., 2003), accumulates ceramide at late developmental stages and shows an early defect in restricting *Botrytis* germination and growth (Bi et al., 2014). Mutation of inositolphosphorylceramide synthase (IPCS), an enzyme that converts ceramide to inositolphosphorylceramide, caused increased accumulation of salicylic acid and ceramide, and enhanced resistance to biotrophic pathogens (Wang et al., 2008). The ceramide synthases encoded by *LAG ONE HOMOLOGUE* (*LOH*) *1*, *LOH2*, and *LOH3* are responsible for the synthesis of ceramides and have different substrate preferences (Markham et al., 2011; Ternes et al., 2011).

Ceramidases degrade ceramides to sphingosine and fatty acids and regulate sphingolipid homeostasis in mammalian and yeast cells. Ceramidases can be divided into three categories: acidic, neutral, and alkaline ceramidases, based on their different catalytic pH values (Mao and Obeid, 2002). Acidic ceramidase mainly occurs in the lysosome, where it is responsible for the decomposition of ceramide (Monjusho et al., 2003). This enzyme has been identified and its full-length cDNA has been cloned from human (Koch et al., 1996) and mice (Li et al., 1998). A deficiency in human acidic ceramidase results in lysosomal storage disorders, commonly known as fat granulomatous disease (Koch et al., 1996). Neutral and alkaline ceramidases affect signal transduction and cell metabolism (Ohlsson et al., 2007). The alkaline ceramidase identified from *Saccharomyces cerevisiae* has a dual activity, catalyzing both hydrolysis and synthesis of yeast ceramide (Mao et al., 2000). Humans also have three alkaline ceramidases (Mao et al., 2001; Xu et al., 2006; Sun et al., 2007). Human alkaline ceramidase 2 can inhibit the synthesis of the subunit of bata1-integrin, thus affecting cell adhesion (Sun et al., 2009). In mammals, neutral ceramidase has been isolated from different tissues, including mouse liver (Tani et al., 2000), kidney (Mitsutake et al., 2001), brain (El Bawab et al., 1999), and intestine (Olsson et al., 2004), and from human intestinal tissue (Ohlsson et al., 2007). In other organisms, neutral ceramidase was also isolated from *Dictyostelium discoideum* (Monjusho et al., 2003), *Drosophila melanogaster* (Yoshimura et al., 2002), and zebra fish (Yoshimura et al., 2004). Although mice have no obvious phenotypic defects when the neutral ceramidase is mutated, they have defects in lipid metabolism in the intestine and increased susceptibility to intestinal cancer (Schmelz et al., 2001; Symolon et al., 2004), indicating that the neutral ceramidase affects ceramide and sphingosine levels in the gastrointestinal tract by regulating dietary sphingolipid metabolism (Kono et al., 2006).

In plants, no homologs of acid ceramidase have been identified, but the *Arabidopsis thaliana* genome encodes one alkaline ceramidase *At*ACER (At4G22330) and three neutral ceramidases, *At*NCER1 (At1g07380), *At*NCER2 (At2g38010), and *At*NCER3 (At5g58980). A neutral ceramidase (*Os*CDase) was cloned from rice (*Oryza sativa* sp. *japonica* cv. Nipponbare). *Os*CDase localizes to the endoplasmic reticulum (ER) and Golgi and appears to use ceramide instead of phytoceramide as a substrate *in vitro* (Pata et al., 2008). Expression of *Ta*-CDase, a wheat neutral ceramidase, could be induced by fungal infection (Yu et al., 2011). However, there is no further investigation about the role of neutral ceramidase in regulation of sphingolipid homeostasis.

Here, we characterized *Arabidopsis* ceramidase 1 (*At*NCER1), a homolog of human neutral ceramidase. Our results demonstrate that *At*NCER1 affects sphingolipid homeostasis and plays a role in response to oxidative stress.

## Materials and Methods

### Mutant Analysis

*Arabidopsis thaliana* wild-type plants Columbia (Col-0) were used in this study and grown in a 16 h light/8 h dark cycle, as described previously (Bi et al., 2014). T-DNA insertion mutant seeds (SALK_054725, designated *ncer1*; SALK_020682, designated *ncer3*) were ordered from ABRC seed stock center (www.arabidopsis.org/). The primers used were: *ncer1*-LP, 5′-TCTCCACCAGTGTAAACGTCC-3′, *ncer1*-RP, 5′-TTTTCATTCTCAGCGTTCCTG-3′, *ncer3*-LP: 5′-TCTATCAGCTCCAGCAAATGG-3′, *ncer3*-RP: 5′-AGTAACGAGGATGCCATTTCC-3′, LBb1.3: 5′-ATTTTGCCGATTTCGGAAC-3′. The double mutant *ncer1 ncer3* was also characterized.

### Plasmid Construction and Subcellular Localization

To generate the *GFP: AtNCER1* construct, we amplified the full-length *AtNCER1* open reading frame from cDNA without the stop codon, using the following primer pair, which includes an *Asc*I restriction site: F (5′-TTGGCGCGCCATGGAGCTATCTCTAGTCAGATTAT-3′) and R (5′-TTGGCGCGCCGTGTTACAACGAAAGCACTAGAA-3′). After *Asc*I digestion, The PCR product was subcloned in frame with EGFP in a vector derived from pUC18. The P35S:eGFP:NCER1 plasmid was co-expressed with a plasmid expressing an ER marker (CD3-960), by transient expression in protoplasts. Protoplast transfection was performed as described previously (Bi et al., 2014). The transfected protoplasts were cultured under weak light (about 300 lux) for 16–24 h at room temperature and observed by confocal microscopy (LSM-780, Carl Zeiss). The excitation/emission wavelengths were: 488 nm/500–530 nm for green fluorescent protein (GFP), 561 nm/580–630 nm for mCherry, and 488 nm/650–750 nm for chlorophyll.

### Methyl Viologen Treatment

Surface-sterilized seeds were sown on ½ MS and grown vertically under 16 h light /8 h dark conditions in an incubator for 7 days and then transferred to half-strength Murashige and Skoog (½ MS) medium containing 1 μM methyl viologen (MV). Samples were collected at 7 days after treatment and extracted and analyzed for sphingolipid profiling.

### Sphingolipid Analysis

Measurement of sphingolipids was performed and analyzed by Shimadzu UFLC-XR (Shimadzu, Japan) coupled with a hybrid quadrupole time-of-flight mass spectrometer (AB SCIEX Triple TOF 5600^+^, Foster City, CA, USA) using Phenomenex Luna C8 column (150 mm × 2.0 mm, 3 μm). Briefly, 30 mg of lyophilized samples was homogenized. The internal standards (C17 base D-erythro-sphingosine and C12-Ceramide) were added and extracted with the isopropanol/hexane/water (55:20:25 v/v/v) and incubated at 60°C for 15 min. After centrifugation, the supernatants were dried and de-esterified in methylamine in ethanol/water (70:30 v/v) as described previously (Wu et al., 2015). The sphingolipid species were analyzed using the software Multiquant (AB SCIEX).

### Quantitative RT-PCR Analysis

Total RNA was extracted with E.Z.N.A. plant RNA kit (R6827-01, Omega Bio-tek). For each sample, 1 μg RNA was reverse transcribed into cDNA using PrimeScript^®^ RT reagent kit (TAKARA, DRR047A). Real time PCR was performed with SYBR Premix Ex Taq || kit (Takara, RR820L) according to the manufacturer’s instructions, and quantitatively analyzed by StepOnePlus^TM^ real-time PCR systems (AB SCIEX). *ACT2* expression level was used as the internal control. All experiments were repeated at least three times. We used the 2^−ΔΔCT^ method (Livak and Schmittgen, 2001) to determine the relative expression level of target genes according to the expression level of *ACT2*.

## Results

### Neutral Ceramidase Mutant and Overexpressing Plants Show Normal Growth and Development

**FIGURE 1:**
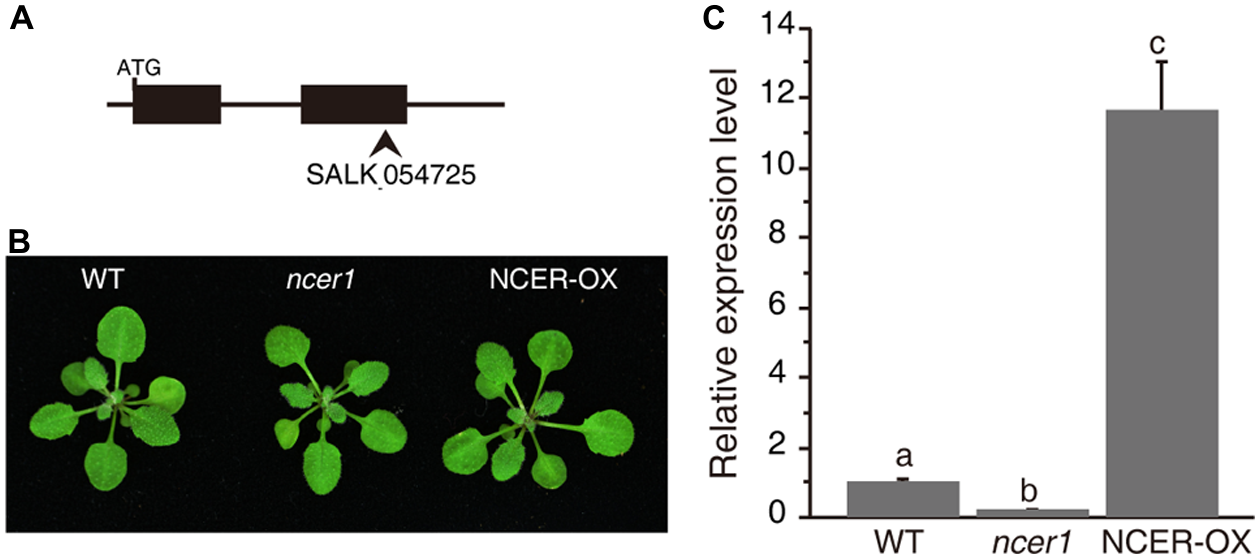
**Phenotypes of the neutral ceramidase mutants. (A)** Gene structure of *AtNCER1*. Rectangles show exons and the arrowhead indicates the T-DNA insertion site. **(B)** Phenotype of 3-week-old wild type, *ncer1*, and *AtNCER1* over-expression line (NCER-OX). **(C)** Real time PCR qualification of *AtNCER1* transcript levels in 3-week-old *ncer1* and NCER-OX plants. *ACT2* was used as the internal control. Gene expression values are presented relative to average wild-type levels, and error bars indicate SE from three technical replicates. This experiment was repeated three times with similar results. Data sets marked with different letters indicate significant differences assessed by a Student’s Newman–Keulst test (*p* < 0.05).

To examine neutral ceramidase function, we first characterized the effect of loss or increase of *At*NCER function. For loss of *At*NCER function, we examined an *AtNCER1* mutant (designated *ncer1*). The *ncer1* mutant has a T-DNA insertion in the second exon of *AtNCER1* (**Figure 1A**). To increase *AtNCER* function, we also generated transgenic plants with a *35S:AtNCER* construct (NCER-OX). Both *ncer1* and NCER-OX plants showed no apparent developmental differences from wild type (**Figure 1B**). However, the expression level of *AtNCER1* reduced by 80% in *ncer1* mutants and increased 12-fold in NCER-OX plants, when compared to wild-type plants (**Figure 1C**).

### *At*NCER1 Localizes on the ER

**FIGURE 2:**
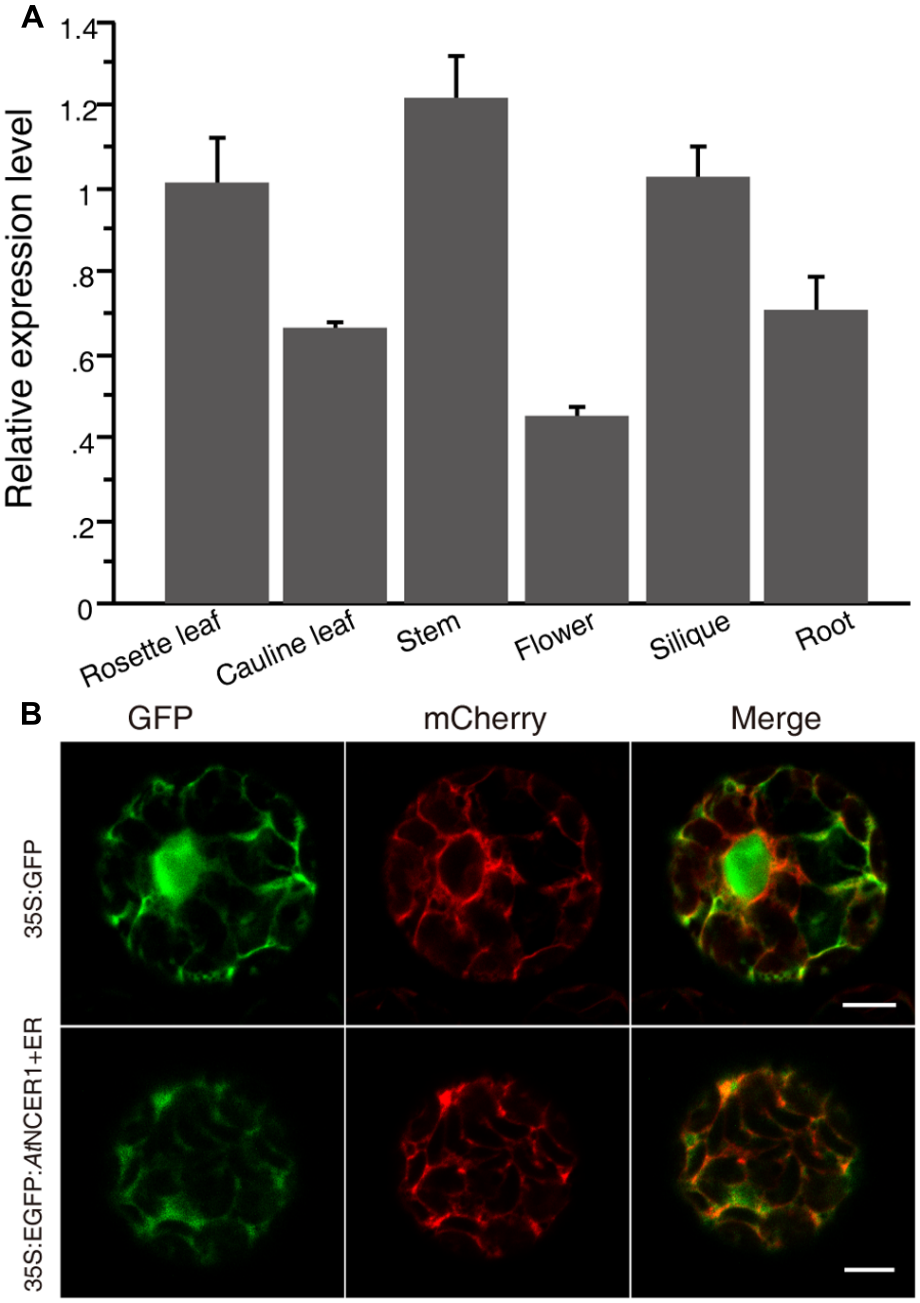
**Expression of *AtNCER* and subcellular localization of *At*NCER. (A)** Expression of *AtNCER1* in different tissues. Reverse transcription quantitative PCR was used to measure the relative transcript levels of *AtNCER1* in the indicated tissues. The transcript level of *ACT2* was used as the internal control. Gene expression values are presented relative to the rosette average leaf level, which was set as 1, and error bars indicate SE. **(B)** Subcellular localization of *At*NCER1. The fusion construct *35S:eGFP:AtNCER1* was co-expressed with the endoplasmic reticulum (ER) mCherry marker (TAIR, CD3-960) by transient expression in protoplasts. The *35S:GFP* construct and the ER mCherry marker was transformed as a control. The images were photographed by confocal microscopy after 16 h incubation. The experiments were repeated at least three times with similar results. Bar = 5 μm.

To examine the expression of *AtNCER1*, we used real-time PCR to quantify its transcript levels in flower, rosette leaf, cauline leaf, stem, silique, and root. *AtNCER1* was expressed in all detected tissues, with relatively low expression in flowers (**Figure 2A**).

To investigate the subcellular localization of NCER1, we constructed a protein fusion to GFP, by constructing a *35S:eGFP:AtNCER1* expression cassette for transient expression in protoplasts with ER-mCherry as a marker. We observed that the GFP fluorescence co-localized with the ER marker, indicating that *At*NCER1 is localized on the ER (**Figure 2B**).

### Neutral Ceramidase Mutants have Increased Sensitivity to C2-Ceramide Induced Cell Death

**FIGURE 3:**
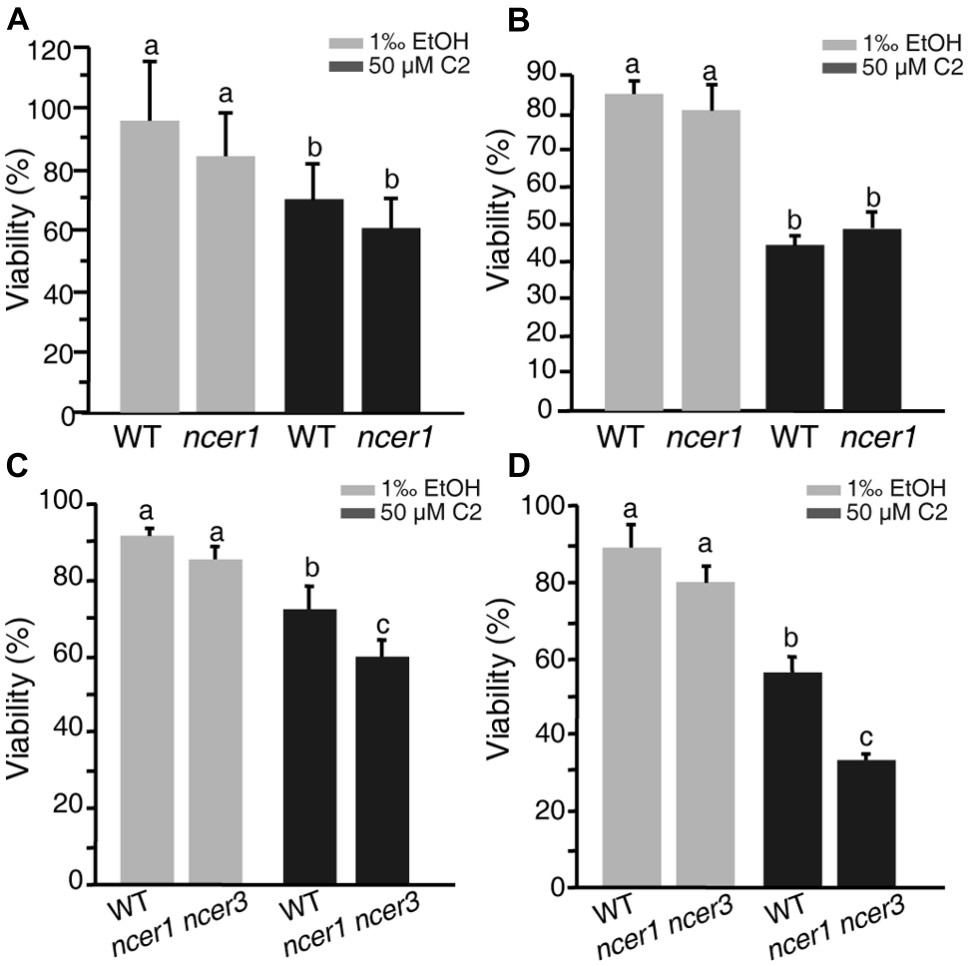
**C2-ceramide treatments.** Protoplasts isolated from 3-week-old wild-type, *ncer1* mutant, and *ncer1 ncer3* double mutant plants were treated with 50 μM C2-ceramide for 24 h **(A,C)** or 48 h **(B,D)**. One thousandth of ethanol was used as the solvent control. Protoplast viability was measured by staining with fluorescein diacetate. Letters indicate statistically different values using a Student’s Newman–Keulst test (*p* < 0.05). For each treatment, at least 500 protoplasts were counted. Error bars indicate SD from three independent experiments.

C2-ceramide is a synthetic short-chain ceramide that can permeate cell membrane and induce cell death (Liang et al., 2003). Ceramidases catalyze hydrolysis of ceramides to generate sphingosine (Mao and Obeid, 2002; Wu et al., 2015). To investigate the sensitivity of *ncer1* mutants to ceramide treatment, we isolated protoplasts from wild-type and *ncer1* plants and treated them with 50 μM C2-ceramide. After 24 h C2 treatment, the viability of protoplasts in *ncer1* remained at 80%, and showed no significant difference from wild type (**Figure 3A**). This trend was maintained to 48 h when the viability of both wild-type and *ncer1* protoplasts decreased to about 40% (**Figure 3B**).

Since *Arabidopsis* has three ceramidase-like genes, *AtNCER1, AtNCER2,* and *AtNCER3*, we therefore identified a T-DNA insertion mutant of *AtNCER3* (designated *ncer3*) and crossed *ncer1* and *ncer3* plants to obtain double mutants. At 24 h, the viability of protoplasts from the double mutants significantly decreased when compared to wild type (**Figure 3C**). At 48 h, double mutant protoplasts showed clear sensitivity to ceramide treatment, compared with wild type (**Figure 3D**). These results indicate that *AtNCER1* and *AtNCER3* may function redundantly in *Arabidopsis.*

### Alteration of Sphingolipids in *ncer1* Mutants

**FIGURE 4:**
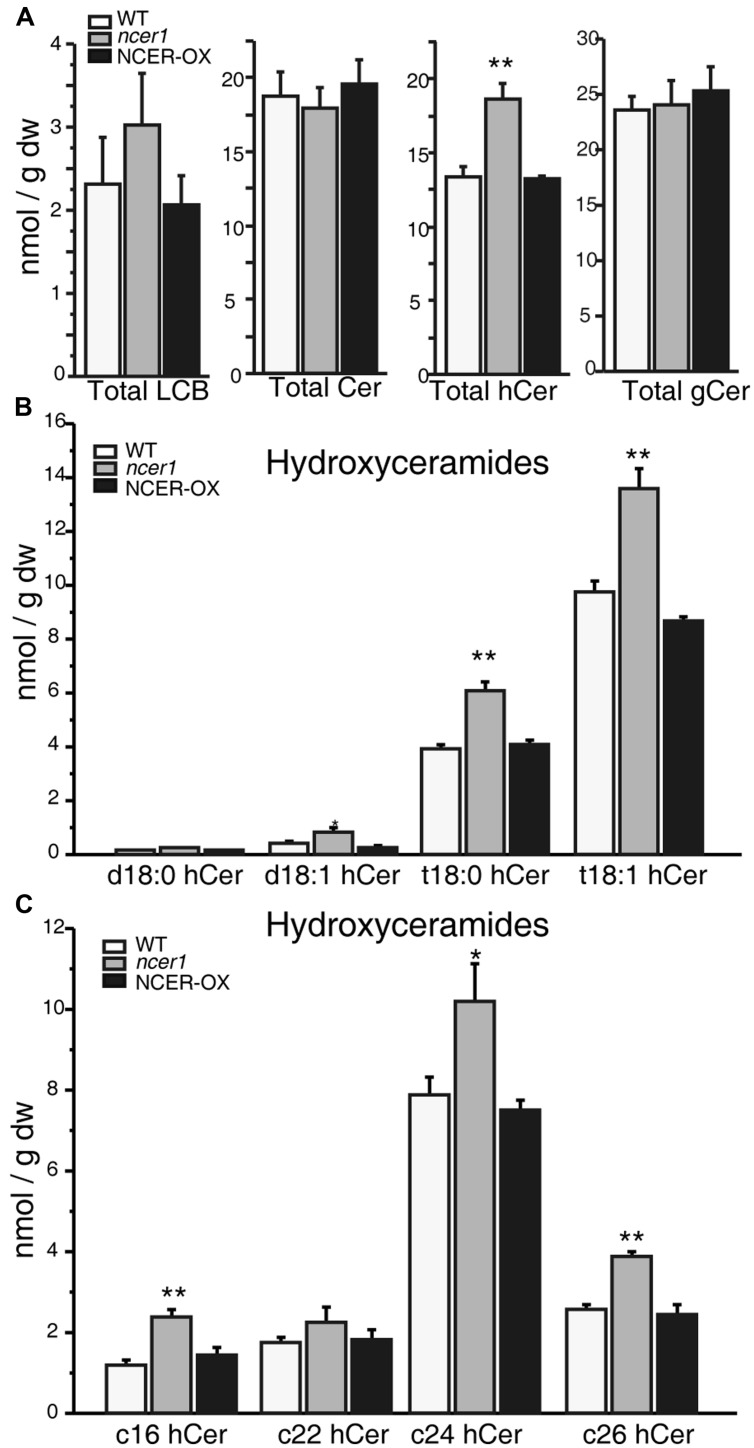
**Sphingolipid analysis in *ncer1* mutant plants.** Sphingolipids were extracted from 21-day-old seedlings as described in Section “Materials and Methods.” The contents of free long chain bases (LCBs), ceramides (Cer), hydroxyceramides (hCer), and glucosylceramides (gCer) were quantified. The experiment was repeated at least three times using independent samples. Asterisks show a significant difference from the wild type using Student’s *t*-test (*p < 0.05, **p < 0.01). **(A)** Total LCBs, ceramides, hydroxyceramides, and glucosylceramides in the indicated plants. **(B)** Comparison of hydroxyceramides with the LCB moieties in the indicated plants. **(C)** Comparison of hydroxyceramides with the length of fatty acid moieties in the indicated plants.

To determine whether the sphingolipid profile changed in *ncer1*, we compared the sphingolipid contents of *ncer1* and NCER-OX with wild type. We found that the *ncer1* mutants showed significant increases in hydroxyceramides, but not ceramides, and no obvious changes in other sphingolipids, including the long chain bases (LCB), ceramides, and glucosylceramides (**Figure 4A**). Comparing hydroxyceramide with LCB moieties, we found a higher level of t18:0 and t18:1 hydroxyceramides in *ncer1* than in wild type (**Figure 4B**). Comparing hydroxyceramides with different length fatty acid moieties, both hydroxyceramide-containing long chain (C16) and very long chain fatty acids (C24 and C26) accumulated (**Figure 4C**). No significant differences of sphingolipid contents were found between wild-type and NCER-OX plants (**Figure 4**). These results indicated that *At*NCER1 may use hydroxyceramides other than ceramide for its substrate.

### Sensitivity of *ncer1* to Oxidative Stress Induced by MV Treatment

**FIGURE 5:**
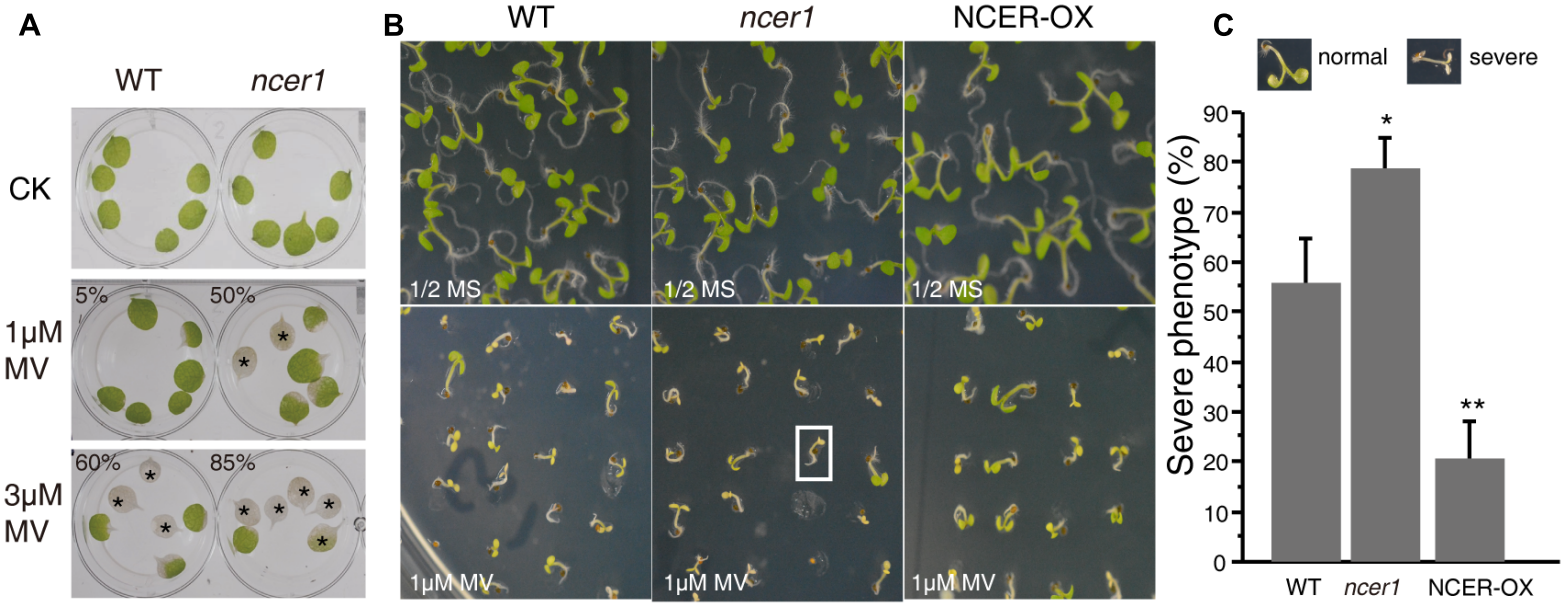
**Sensitivity of *ncer1* mutants to oxidative stress induced by MV. (A)** Disks from the third and fourth leaves of 14-day-old wild-type and *ncer1* mutant plants were incubated with or without 1 μM or 3 μM MV for 3 days and then photographed. Asterisks indicate completely bleached, damaged leaf disks. At least 12 leaves of wild-type and *ncer1* were observed in each experiment. Two independent tests were done. The ratio of the bleached area and the whole leaf area showed significantly difference from the wild type using Student’s *t*-test (*p* < 0.05). **(B)** The phenotype of wild type, *ncer1*, and NCER-OX seedlings after MV treatment. Seedlings were grown on 1/2 MS containing or without 1 μM MV for 10 days and then photographed. The white square indicates one seedling with a severe phenotype. **(C)** Percentage of seedlings with a severe phenotype after 1 μM MV treatment. At least 250 seedlings were counted in each genotype. Error bars indicate SD from three independent experiments. Asterisks indicate significant differences by Student’s *t*-test (p* < 0.05; **p < 0.01).

Reactive oxygen species (ROS) play an important role in the programmed cell death induced by ceramides (Bi et al., 2014). To examine oxidative stress tolerance in wild-type and mutant plants, we treated detached leaves with MV. After 3 days of treatment, we found significantly more leaves from *ncer1* plants became bleached in both 1 and 3 μM MV treatments, compared with wild-type, indicating that the *ncer1* mutants are more sensitive to oxidative stress (**Figure 5A**). We also performed a plate assay, in which surface-sterilized seeds were sown on 1/2x MS agar plates containing 1 μM MV and incubated for 10 days (**Figure 5B**). We found that about 80% of *ncer1* mutant seedlings lost their green color and showed severe damage on plates containing 1 μM MV, compared with 55% of wild-type seedlings. Strikingly, NCER-OX seedlings showed increased tolerance to 1 μM MV (**Figure 5C**). These results showed that *AtNCER1* affects *Arabidopsis* sensitivity to MV-induced oxidative stress.

**FIGURE 6:**
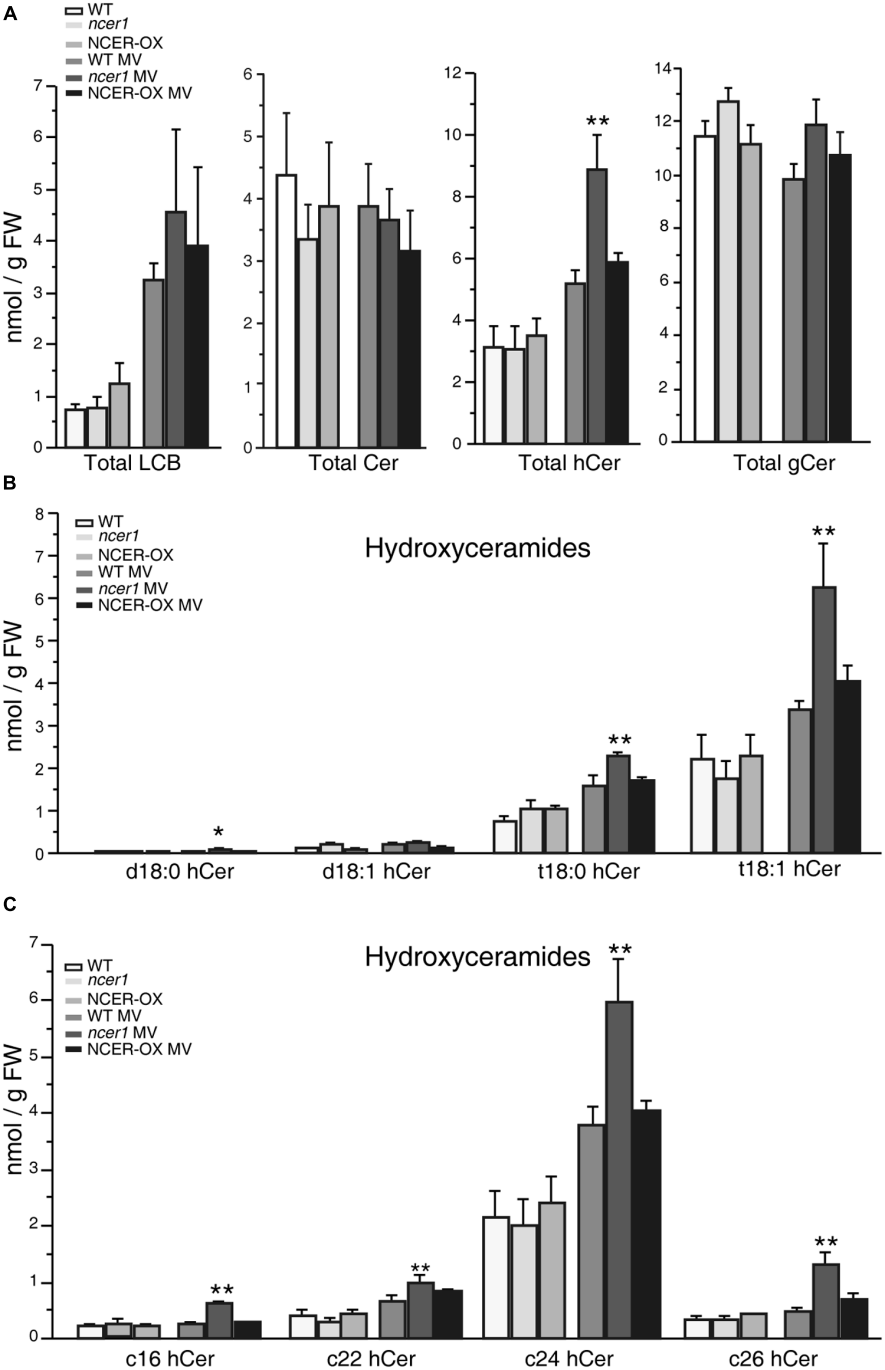
**Sphingolipid analysis after MV treatment.** Seven-day-old seedlings were transferred to 1/2x MS containing 1 μM MV. Sphingolipids were extracted after 7 days with or without MV treatment, as described in Section “Materials and Methods.” The contents of free LCBs, ceramides (Cer), hydroxyceramides (hCer), and glucosylceramides (gCer) were quantified. The experiment was repeated three times using independent samples. Data sets marked with asterisks indicate significant differences confirmed by Student’s *t*-test (*p < 0.05; **p < 0.01). **(A)** Total LCBs, ceramides, hydroxyceramides, and glucosylceramides after MV treatment. **(B)** Comparison of hydroxyceramides with the LCB moieties in the indicated plants after MV treatment. **(C)** Comparison of hydroxyceramides with the length of fatty acid moieties in the indicated plants after MV treatment.

To test whether MV oxidative stress affects the sphingolipid profile, we transferred 7-day-old seedlings to 1/2x MS agar plates containing 1 μM MV, then collected samples after 7 days. No significant difference in sphingolipid contents was shown in untreated control plants at early developmental stages (**Figure 6**). Compared with untreated controls, the amount of total LCB and hydroxyceramide increased significantly in response to MV treatments; also, *ncer1* showed almost threefold more LCB and hydroxyceramide than non-treated control plants (**Figure 6A**). No significant changes were observed in the ceramides and glucosylceramides (**Figure 6A**). The amounts of hydroxyceramides containing both long chain and very long chain fatty acids showed significant accumulation in *ncer1* compared with wild-type plants (**Figure 6C**). The wild-type and NCER-OX plants showed no significant differences in sphingolipid contents (**Figure 6**). A significantly high level of t18:0 and t18:1 hydroxyceramides accumulated in *ncer1* mutants, compared with wild type, and NCER-OX plants, after MV treatments (**Figure 6B**), indicating that hydroxyceramides may be a potential substrate of *At*NCER1.

## Discussion

Sphingolipids are important signaling molecules involved in the regulation of cell physiological activities and in the mediation of plant responses to biotic and abiotic stress (Dickson and Lester, 2002; Hannun and Obeid, 2002; Spiegel and Milstien, 2003; Futerman and Hannun, 2004;Pyne et al., 2009). Plants have about 200 different kinds of sphingolipid molecules (Chen et al., 2009), with high diversity in the composition of the head groups, the degree of hydroxylation, and the position of the double bond (Chen et al., 2008). Sphingolipids have four hydroxylation sites: the C-1 and C-3 hydroxyl groups arise from Ser and palmitoyl-CoA precursors, respectively, and the third hydroxyl group at the C-4 position is synthesized by LCB C-4 hydroxylases encoded by *SPHINGOID BASE HYDROXYLASE 1* (*SBH1*) and *SBH2*. The *sbh1/2* mutants have a severely dwarfed phenotype and fail to progress from vegetative to reproductive growth, which may result from the accumulation of sphingolipids with the dihydroxy LCB/C16 fatty acid–containing ceramide backbone (Chen et al., 2008). The *Arabidopsis thaliana* genome harbors two *FATTY ACID HYDROXYLASE* genes (*AtFAH1* and *AtFAH2*), which encode the enzymes that catalyze α-hydroxylation of the fatty acid moiety in plant sphingolipids. The *fah1*/*fah2* mutants showed elevated levels of LCBs and ceramides and resistance to *G. cichoracearum* infections (Konig et al., 2012). These data suggest that sphingolipid hydroxylation has a very important role in regulation of the plant response to biotic and abiotic stress.

Few studies have examined the connection between elevated hydroxyceramides and induction of cell death in plants. However, in many mammalian cell types, evidence clearly shows that hydroxyceramide and hydroxy complex sphingolipids have unique functions in membrane homeostasis and cell signaling, and these functions cannot be substituted by their non-hydroxy counterparts (Kota and Hama, 2014). Trihydroxy LCBs (tLCBs) of 4-hydroxysphinganine, t16:0, t18:0, t19:0, and t20:0, and non-hydroxy fatty acids (NFAs) isolated from equine kidneys exhibited stronger apoptosis-inducing activities than dLCB-NFA toward tumor cell lines (Kyogashima et al., 2008). In plants, we previously found that *acd5* mutants show a modest increase in the amount of hydroxyceramides, relative to wild type, even in 17-day-old plants. In addition, after *Botrytis cinerea* infections, hydroxyceramides in *acd5* increased to a greater extent than in wild-type plants (Bi et al., 2014). These results suggest that hydroxyceramides are involved in programmed cell death and biotic stress. In this work, we found that total LCB levels increased after MV treatment, but found no significant difference between wild type and mutants. By contrast, without treatment, 3-week-old *ncer1* plants accumulated more hydroxyceramide compared with wild type. Furthermore, when challenged by MV treatment, hydroxyceramide increased to higher levels in *ncer1* plants than in wild type, indicating that hydroxyceramides may be involved in oxidative stress.

A neutral rice ceramidase appears to use ceramide instead of phytoceramide as a substrate in the CDase mutant stain of yeast (Pata et al., 2008). Our results indicated *AtNCER1* may favor hydroxyceramides as its substrates. Our future work will look for potential AtNCER1 substrates, especially t18:0 and t18:1 hydroxyceramides.

Ceramide, as a bioactive lipid key player, involves in various cellular processes (Hannun and Obeid, 2002; Chen et al., 2009). Recent studies have demonstrated that sphingolipids can trigger the generation of ROS in both mammalian and plant cells (Hannun and Obeid, 2002; Shi et al., 2007). Certain ceramides can directly inhibit the activity of mitochondrial complex IV, leading to ROS production and oxidative stress (Kogot-Levin and Saada, 2014). In this work, we show that *AtNCER1* regulates the plant response to oxidative stress. We speculate that the turbulence of sphingolipid in *ncer1* may change intracellular redox state or effect of antioxidant systems. Further study may provide insights into the understanding of interplay between ROS and sphingolipids.

## Conclusion

In this report, we characterized an *Arabidopsis* neutral ceramidase mutant (*ncer1*). Our results indicate that *ncer1* accumulated high levels of hydroxyceramides in normal conditions, but showed no visible phenotype. We also found that *ncer1* mutants were more sensitive to oxidative stress induced by MV and had higher levels of hydroxyceramides, indicating that *AtNCER1* regulates the plant response to oxidative stress. Further study may provide insights into the understanding of interplay between ROS and sphingolipids.

## Conflict of Interest Statement

The authors declare that the research was conducted in the absence of any commercial or financial relationships that could be construed as a potential conflict of interest.
